# A short guide to the tight junction

**DOI:** 10.1242/jcs.261776

**Published:** 2024-05-07

**Authors:** Sandra Citi, Michael Fromm, Mikio Furuse, Lorenza González-Mariscal, Asma Nusrat, Sachiko Tsukita, Jerrold R. Turner

**Affiliations:** ^1^ Department of Molecular and Cellular Biology, University of Geneva, 30 Quai Ernest Ansermet, 1205 Geneva, Switzerland; ^2^ Clinical Physiology/Nutritional Medicine, Department of Gastroenterology, Charité – Universitätsmedizin Berlin, Campus Benjamin Franklin, Hindenburgdamm 30, 12203 Berlin, Germany; ^3^ Division of Cell Structure, National Institute for Physiological Sciences, 5-1 Higashiyama Myodajii, Okazaki 444-8787, Japan; ^4^ Department of Physiology, Biophysics and Neuroscience, Center for Research and Advanced Studies (CINVESTAV), Av. Instituto Politécnico Nacional 2508, Mexico City 07360, México; ^5^ Mucosal Biology and Inflammation Research Group, Department of Pathology, University of Michigan, 109 Zina Pitcher Place, 4057 Biomedical Science Research Building, Ann Arbor, MI 48109-2200, USA; ^6^ Advanced Comprehensive Research Organization (ACRO), Teikyo University, Kaga 2-21-1, Itabashi-ku, Tokyo 173-0003, Japan; ^7^ Laboratory of Mucosal Barrier Pathobiology, Department of Pathology, Brigham and Women's Hospital, Harvard Medical School, 77 Avenue Louis Pasteur, Boston, MA 01125, USA

**Keywords:** Actin, Claudin, Epithelium, Myosin, Permeability, Tight junctions, ZO-1, Cingulin, Occludin, Barrier, Polarity

## Abstract

Tight junctions (TJs) are specialized regions of contact between cells of epithelial and endothelial tissues that form selective semipermeable paracellular barriers that establish and maintain body compartments with different fluid compositions. As such, the formation of TJs represents a critical step in metazoan evolution, allowing the formation of multicompartmental organisms and true, barrier-forming epithelia and endothelia. In the six decades that have passed since the first observations of TJs by transmission electron microscopy, much progress has been made in understanding the structure, function, molecular composition and regulation of TJs. The goal of this Perspective is to highlight the key concepts that have emerged through this research and the future challenges that lie ahead for the field.

## Teaser

**Summary:** We summarize the key structural and functional features of vertebrate tight junctions, and the mechanisms and regulation of the tight junction barrier in physiology and pathology.

## Introduction

Tight junctions (TJs) are identified on the basis of their unique combination of ultrastructural and functional characteristics (Farquhar and Palade, 1963; Staehelin, 1974; Diamond, 1977; see Box 1). Structurally, TJs are formed by a continuous mesh-like network of strands surrounding the apex of epithelial cells that connects and tightens the space between neighboring cells (Farquhar and Palade, 1963; Staehelin, 1974). Functionally, TJs seal the paracellular pathway in a highly specialized manner, either by forming a barrier against the passage of most solutes (‘tight’ epithelia) or by allowing the passage of ions and water through paracellular channels (‘leaky’ epithelia) (Claude and Goodenough, 1973; Van Itallie and Anderson, 2014; Buckley and Turner, 2018; Van Itallie, 2018). In the past four decades, the molecular basis of TJ structure and function has been clarified. The TJ barrier is constituted by the assembly of different specific transmembrane proteins on the plasma membranes of adjacent cells, featuring side-by-side cis interactions as well as trans interactions (Tsukita et al., 2019; Otani and Furuse, 2020; Piontek et al., 2020). TJ transmembrane proteins are connected intracellularly with complexes of scaffolding and adaptor proteins, which are in turn associated directly or indirectly with the actomyosin and microtubule cytoskeletons. Both the scaffolding and adaptor proteins and the cytoskeleton perform architectural and regulatory functions (Yano et al., 2017; Rouaud et al., 2020; Wibbe and Ebnet, 2023). Overall, this molecular organization allows TJs to establish and maintain a separation between internal, luminal and exterior environments, and also to function as polarity and signaling hubs. Importantly, a variety of diseases have been linked to altered TJ structure and composition and, hence, disrupted barrier function (Buckley and Turner, 2018).

**Box 1. The identification and characteristics of TJs**

Molecular components of TJs can be detected at cell–cell contact sites and the cell periphery using immunofluorescence microscopy. However, this localization is not sufficient to identify a TJ, since several TJ proteins, including claudin family members, are detected not only at TJs but also along non-junctional contact sites of polarized epithelial cells, as well as in cells that do not form TJs. For example, ZO-1 is not only associated with epithelial and endothelial TJs but is also expressed in non-epithelial cells and tissues that lack TJs (for example cardiac myocytes), where it is localized at AJs. Certain TJ proteins, such as cingulin and occludin, appear to be more specific markers for TJs, but they can be detected elsewhere in specific contexts (for example, cingulin is localized in the apical cortex of frog oocytes). Therefore, TJs cannot be defined exclusively based on immunofluorescence microscopy-based observations of the peripheral localization of known TJ proteins, unless the following ultrastructural, functional, architectural and molecular criteria are also fulfilled.

**Ultrastructural criteria**

(1) Intimate apposition of outer membrane leaflets (seen by TEM).

(2) Claudin-based strands or fibrils (seen by FFEM).

(3) Central tube (in tTJs).

**Functional criteria**

(1) Barrier function (tight to leaky).

(2) Selective permeability.

**Architectural criteria**

(1) Continuous circumferential ring along the borders of adjoining cells in a sheet.

(2) Apical to AJs.

**Molecular criteria**

(1) Transmembrane proteins: claudins, Ig-like CAMs and TAMPs.

(2) Cytoplasmic scaffolding and adaptor proteins (ZO proteins, cingulin).

(3) Connection to actomyosin and microtubule cytoskeletons.

At a 2023 meeting in Leysin, Switzerland, a wide range of themes in TJ research were discussed, ranging from the structure and organization of TJ protein components to the role of TJs in cell biology, development, physiology and pathology. Here, based on discussions at the meeting and on many decades of studies of TJs since their first description, we provide a short guide to the main structural, functional, architectural and molecular features of TJs, integrating the most recent findings and concepts, and highlighting challenges and open questions for future research.

## The ‘zonula occludens’ of epithelial cells

The ‘canonical’ TJ (or zonula occludens, referred to hereafter as ZO) was first described by Farquhar and Palade following transmission electron microscopy (TEM) analysis of vertebrate polarized epithelial intestinal cells (Farquhar and Palade, 1963). The TJ is the most apical intercellular junction and is located immediately above the adherens junction (AJ), which is called the zonula adhaerens (ZA) in polarized epithelial cells (Meng and Takeichi, 2009). The term ‘zonula’ refers to a thin and continuous circumferential belt. The term ‘occludens’ refers to the ability of TJs to occlude (that is, close or seal) the intercellular space, as seen in transmission electron micrographs of TJs*.* The term ‘adhaerens’ refers to the main function of the ZA, which is to maintain adhesion between neighboring epithelial cells, supporting the formation of continuous cell sheets.

Both ZOs and ZAs form continuous belts that encircle the apical regions of epithelial cells and integrate individual cells into a tissue. The resulting epithelial continuity is required for barrier function, which must be maintained throughout development and the life cycle of cells and tissues. This is accomplished by homeostatic remodeling of TJs during cell proliferation, migration, differentiation and extrusion. The critical need for preservation of barrier function, which protects underlying tissue compartments from pathogens, toxins and other insults, is emphasized by the presence of specialized repair mechanisms, such as RhoA GTPase activation at sites of local ZA and ZO discontinuities (Higashi et al., 2016; Citi, 2019). Thus, despite the apparently uniform appearance of TJs, these structures are highly dynamic, and repair mechanisms exist to restore the barrier when needed.

TJs and AJs are intimately connected spatially and functionally and are collectively referred to as the apical junctional complex (AJC) (Quiros and Nusrat, 2014; Naser et al., 2023). During epithelial differentiation and apico-basal polarization, TJ and AJ components initially colocalize but are eventually sorted into distinct apical TJs and basal AJs. By providing the structural integrity to maintain epithelial cells in close apposition, the AJ is a prerequisite for the formation and function of the TJ. The TJ forms a seal but lacks the adhesive strength necessary to organize cell apposition. Conversely, the AJ provides the structural integrity that maintains epithelial cells in close apposition but cannot form a seal. Both TJs and AJs are connected, albeit in different manners, to the circumferential actomyosin belt, and they represent a functional unit, as together they affect the structure and function of the AJC.

## The ultrastructure, molecular architecture and remodeling of TJs

**Fig. 1.**
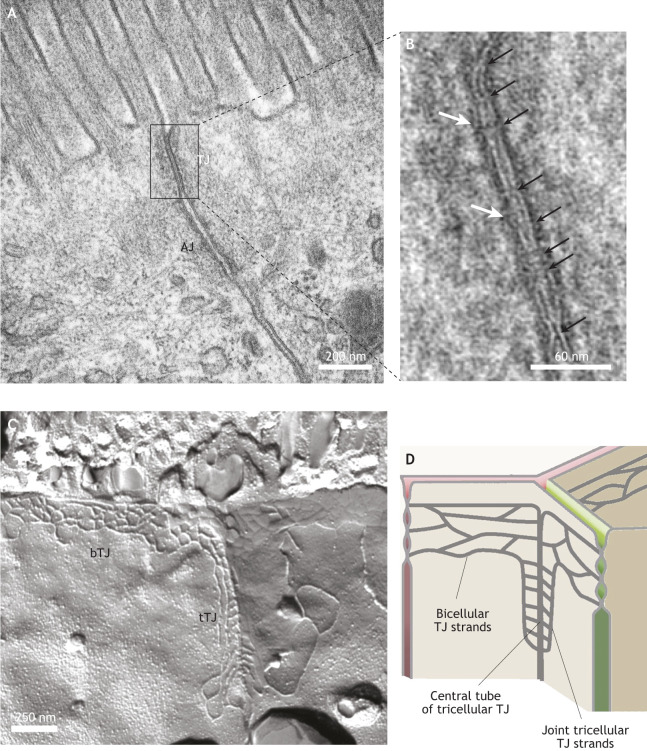
**The ultrastructure of TJs.** (A) Transmission electron micrograph of the AJC of mouse intestinal epithelial cells (within the jejunum), highlighting the position of the apical TJ and the neighboring AJ. (B) Detailed image of the TJ region highlighted in A, with sites of intimate plasma membrane apposition indicated by black arrows, and the electron-dense cytoplasmic plaque immediately beneath the plasma membrane indicated by white arrows. (C) FFEM image of the apical regions of epithelial intestinal (human jejunum) cells, showing the strands of a bTJ and a tTJ. (D) Cartoon depicting bTJ and tTJ structure, highlighting the positions of bTJ strands, joint tTJ strands and the central tube of a tTJ. Images in A and B provided by Kyoko Furuse, National Institute for Physiological Sciences, Okazaki, Japan. Image in C provided by Susanne M. Krug, Charité – Universitätsmedizin Berlin, Germany.

TJs were originally described as focal sites of intimate apposition of outer plasma membrane leaflets, as imaged using TEM (Fig. 1A and black arrows in Fig. 1B), corresponding to a circumferential network of TJ strands or fibrils, as imaged using freeze-fracture electron microscopy (FFEM) (Fig. 1C). The strands are now recognized to be formed by members of the claudin family of tetraspan transmembrane proteins (Piontek et al., 2020). Additional specific TJ transmembrane proteins, such as TJ-associated MARVEL proteins (TAMPs) and immunoglobulin-like cell–cell adhesion molecules (Ig-like CAMs), are important both in barrier function and its regulation, and some can be integrated into strands. Tricellular TJs (tTJs) are sites at which three cells come together. The anatomical ultrastructure and molecular composition exhibited by tTJs are distinct from those of bicellular TJs (bTJs; Fig. 1C and cartoon in Fig. 1D) (Sugawara et al., 2021). For example, tTJs show a central tube and joint tricellular strands that extend more basally with respect to the network of bTJ strands (Fig. 1C,D). The barrier function of tTJs is maintained by tricellulin (also known as MARVELD2, a member of the TAMP family) and angulins (also known as immunoglobulin-like domain-containing receptors, members of the Ig-like CAM family). The precise contribution of lipids to the structural organization of TJs is still unclear. Sphingomyelin and cholesterol are enriched in the plasma membrane fraction that contains TJs and are required for TJ barrier function (Ikenouchi, 2018; Shigetomi et al., 2023). However, the mechanisms by which lipids become sorted and spatially organized at TJs are still unknown.

The cytoplasm underlying the TJ membrane shows less-electron-dense material compared to that observed beneath the AJ (Fig. 1A and white arrows in Fig. 1B), but it nevertheless contains crucially important scaffolding, adaptor and signaling proteins. These include the ZO proteins (ZO-1, ZO-2 and ZO-3; also known as TJP1, TJP2 and TJP3, respectively), cingulin (CGN), guanine-nucleotide-exchange factors (GEFs) and GTPase-activating proteins (GAPs), which not only scaffold TJ membrane proteins but also affect TJ strand formation and dynamics, regulation and repair, by connecting TJs to the cytoskeleton and its regulatory proteins (Wibbe and Ebnet, 2023).

TJs are very dynamic, and their integrity and function are regulated by the trafficking of TJ proteins into and out of cell–cell contacts. In fact, the endocytosis and recycling of TJ proteins plays an important role in steady-state TJ remodeling under homeostatic conditions (Nighot and Ma, 2021). Moreover, excessive internalization of TJ proteins is a major driver of TJ disassembly in disease states associated with compromised epithelial barriers. Several mechanisms of cell surface protein endocytosis have been implicated in the regulation of TJ remodeling and disassembly in normal and disease states, including clathrin-mediated endocytosis, caveolae- and lipid raft-mediated endocytosis, and micropinocytosis (Ivanov et al., 2004).

## TJs in non-epithelial cell types and invertebrate species

TJs also occur in endothelial cells but, because of the very flat shape of these cells, it is difficult to spatially distinguish TJs from AJs in this context, and the two types of junction can be considered as intermixed (Hashimoto and Campbell, 2020). In vascular tissues with limited barrier function, endothelial TJs are often discontinuous and could be described as an AJC, without further distinction into TJ and AJ. However, continuous TJs are present in endothelia at specialized sites including the brain, where they form the blood–brain barrier. Another blood–tissue barrier is the blood–testis barrier, which is formed by Sertoli cells. The TJs formed by Sertoli cells are atypical, because the circumferential TJ strands, which are made by claudin-11, are in the basal rather than apical part of the cells. TJ-like structures are also found in the myelin of the central and peripheral nervous systems (Mitic et al., 2000). For example, Schwann cells of peripheral myelinated axons contain claudin-11 and claudin-19, which are involved in the electrophysiological sealing within each myelinating cell, but not in their morphogenesis (Miyamoto et al., 2005). Axo-glial paranodal junctions instead have an ultrastructure and molecular composition similar to that of septate junctions (Bhat, 2003).

TJ-like structures have been described in invertebrate phyla, where they exhibit a variety of morphologies and architectural and molecular features that distinguish them from vertebrate TJs (Izumi and Furuse, 2014). For example, the septate junctions of insects, which are morphologically categorized as either pleated septate junctions in ectodermally derived cells or smooth septate junctions in endodermally derived cells, show an intercellular space with ladder-like septa instead of a close apposition of neighboring membranes (Izumi and Furuse, 2014). Molecularly, insect pleated septate junctions contain members of the claudin family of proteins, as well as other classes of transmembrane proteins, such as neurexins. Architecturally, insect septate junctions are localized below AJs in polarized epithelia, unlike vertebrate TJs, although there are some exceptions, such as the septate junctions of the *Drosophila* midgut (Chen et al., 2018). Occasional examples of TJs with an ultrastructure similar to that of vertebrate TJs have been described in invertebrates, such as arachnids (Lane, 1992). Furthermore, Porifera and other invertebrate phyla contain claudin-like proteins, underlining the evolutionary importance of claudins in barrier function (Jonusaite et al., 2023). However, the compositional diversity and functional complexity of TJ-like structures in invertebrate phyla are not yet understood.

## TJ barrier function

**Fig. 2.**
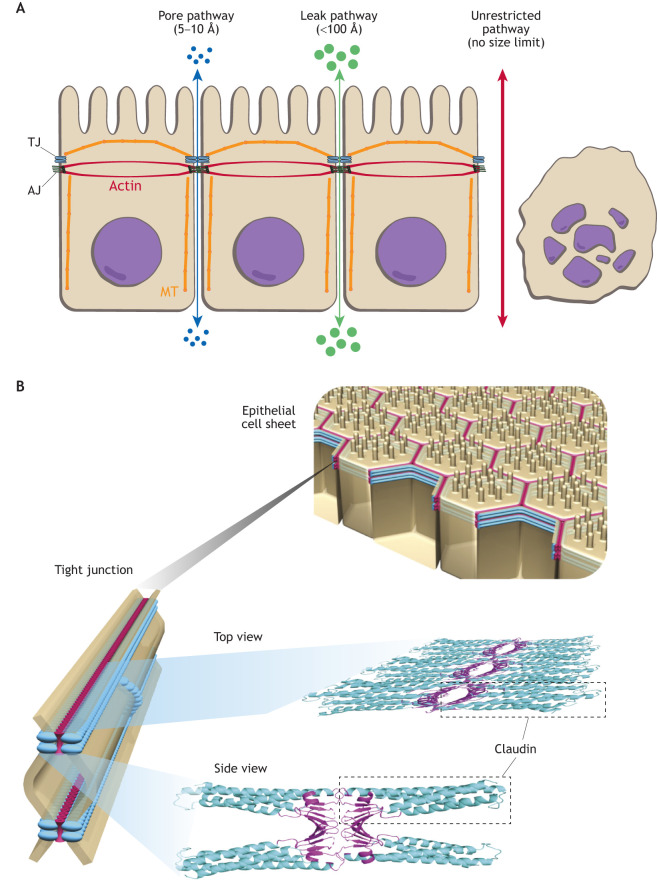
**The barrier function of TJs, and claudin organization and structure.** (A) Schematic of a sheet of polarized epithelial cells, highlighting the position of TJs and AJs. Arrows indicate three functionally defined paracellular pathways: (1) the ‘pore’ pathway formed by claudin-based channels (blue); (2) the ‘leak’ pathway formed by breaks within the bTJ and/or by opening of the tTJ central tube (green); and (3) the ‘unrestricted’ pathway caused by epithelial damage (red). The actin and microtubule (MT) cytoskeletons are outlined in a simplified organization within the cell in red and orange, respectively. (B) Schematic of a sheet of polarized epithelial cells (top), with an expanded section of the TJ region shown (bottom left) alongside top and side views of polymerized claudins within the TJs (bottom right). Note that individual claudin molecules interact in cis and in trans with other claudin molecules in this schematic drawing. Dashed boxes indicate claudin monomers. For more details, see Suzuki et al. (2015).

The main function of TJs is to form a paracellular barrier to the free diffusion of water and solutes of all sizes (ranging from ions to macromolecules and pathogens) across the paracellular space. TJs are therefore able to maintain gradients generated by active transcellular transport. At a few sites, such as the epidermal or bladder epithelia, TJs are nearly impermeable. In essentially all other tissues, TJs are more or less selectively permeable. As a result, transepithelial ion, charge or concentration gradients generated by selectively permeable TJs can also direct passive paracellular flux. Flux across TJs occurs through two main pathways. The ‘pore’ pathway is a high-capacity, charge-selective route for water, small ions and uncharged molecules with a maximum diameter between ∼5 Å and ∼10 Å (Fig. 2A, blue arrow). The pore pathway is formed by claudin channels. These channels are formed extracellularly and not across cell membranes, thus distinguishing them from membrane channels and gap junction channels. The second pathway is the ‘leak’ pathway – a low-capacity, charge-nonselective route that accommodates macromolecules with a maximum diameter of ∼100 Å (Fig. 2A, green arrow), as determined based on the permeability to dextran polymers of different sizes. Complete disruption of the AJC leads to unrestricted flux of solutes across epithelial sheets (‘unrestricted’ pathway; Fig. 2A, red arrow). By definition, ‘leaky’ epithelia have a paracellular pathway with ion conductivity that is higher than that of the transcellular pathway. Conversely, ‘tight’ epithelia exhibit a transcellular pathway with higher ion conductivity than that of the paracellular pathway (Claude and Goodenough, 1973; Van Itallie and Anderson, 2014; Buckley and Turner, 2018; Van Itallie, 2018). Interestingly, in organs that form tubular epithelia – such as the intestine, nephron, and salivary and sweat gland ducts – leaky epithelia are typically found in proximal segments (such as the small intestine and the kidney proximal tubule), whereas tight epithelia are typically present in distal segments (such as the colon and the kidney cortical collecting duct). Leaky epithelia can transport at a high rate but only against minute gradients. Tight epithelia transport at low rate but, if necessary, against high gradients. Impermeable epithelia constitute a small third group and are defined by having a paracellular resistance that is more than 100 times higher than the transcellular resistance and practically no paracellular transport function (Tsukita et al., 2019; Piontek et al., 2020; Shashikanth et al., 2022). Most endothelia have leaky or extremely leaky TJs, with the exception of those constituting blood–tissue barriers, such as the endothelia of brain capillaries, which are tight.

## The molecular basis of TJ permeability

The characteristic TJ strands seen by FFEM are composed of claudin polymers, which are both key components of the barrier and constitute the pore pathway (Fig. 2B) (Tsukita et al., 2019; Otani and Furuse, 2020; Piontek et al., 2020; Meoli and Günzel, 2023). Structural models and molecular dynamics simulations suggest that claudins polymerize in cis to form two parallel rows, which interact in trans with similar polymers on an adjacent cell (Fig. 2B) (Suzuki et al., 2014, 2015; Berselli et al., 2022). Simulations further indicate that the extracellular domains of ‘barrier-forming’ claudins establish the barrier and that tetramers of ‘channel-forming’ claudins generate paracellular pores. The molecular architecture of the pore pathway, consisting primarily of channels formed by channel-forming claudins, has been studied in detail. Claudin-2 forms a channel that primarily accommodates small cations (Na^+^ and Ca^2+^) and water, and the maximum size of molecules that can traverse claudin-2 channels is ∼5.7 Å. The atomic-resolution structures of selected claudins reveal that claudin adopts an outward-facing structure reminiscent of a palm-like β-sheet, which forms a charged surface that can selectively permit the passage of either positive or negative ions through β-barrel-like pores between cells. Functionally, channel-forming claudins can be categorized into three groups, depending on whether they are selective for small cations and water, small cations, or small anions. Whereas most barrier-forming claudins tighten the TJ in a charge-nonselective way, a few build barriers that are differently selective for cations and anions, with most of them being cation barriers (i.e. anion-selective).

The simple classification of claudins as either barrier-forming or channel-forming can be further complicated, considering that interactions between different claudin isoforms suggest that they should be clustered into several distinct groups (Tsukita et al., 2019; Piontek et al., 2020; Meoli and Günzel, 2023). For example, some claudins segregate into separate regions of the strand network, some claudins that cannot form homotypic strands can integrate into a network formed by a different claudin, and some claudins can disrupt a homotypic network formed by another claudin (Gonschior et al., 2022; Shashikanth et al., 2022). There are also additional families of TJ transmembrane proteins that contribute to barrier function. For example, immunoglobulin-like adhesion molecules such as junctional adhesion molecule A (JAM-A, also known as F11R) and coxsackievirus and adenovirus receptor (CAR, also known as CXADR) at bTJs, and angulins at tTJs, are involved in adhesion, barrier function and signaling. Members of the TAMP family (occludin and MARVELD3 at bTJs, and tricellulin at tTJs) have been implicated in controlling barrier function by influencing TJ architecture and morphology (Ikenouchi et al., 2005; Higashi et al., 2013; Luissint et al., 2016; Hartmann et al., 2020; Higashi and Chiba, 2020; Otani and Furuse, 2020). tTJs are particularly important for the leak pathway and are primary targets affected under pathological conditions (Krug et al., 2009, 2014, 2018; Sugawara et al., 2021). Finally, the scaffolding proteins ZO-1 and ZO-2 are redundantly required for barrier assembly, because depletion of both is required to abolish claudin strand assembly (Otani and Furuse, 2020). Despite the major advances in understanding the molecular basis of TJ permeability, many molecular details that govern claudin interactions remain to be clarified. Moreover, the mechanisms by which lipids contribute to TJ barrier assembly and function are still unclear.

## Claudins can be functionally redundant

The claudin family in mammals is encoded by 27 genes that are differentially expressed in a tissue- and species-dependent manner (Tsukita et al., 2019; Piontek et al., 2020; Stamp et al., 2023). In general, multiple claudins are expressed within each cell type, and expression patterns are regulated spatially, temporally, and in response to developmental and pathological stimuli. Furthermore, within certain tissues, such as the intestine and kidney, the complement of expressed claudin proteins changes spatially, for example as a function of either differentiation along the crypt–villus axis or along the nephron segments, respectively (Kiuchi-Saishin et al., 2002; Holmes et al., 2006; Yu, 2015; Meoli and Günzel, 2023). There appears to be significant functional redundancy within the claudin family as, in many cases, knockout (KO) of an individual claudin isoform does not lead to abnormal permeability in cultured cell models or mice (Tokuda et al., 2014). One of the few exceptions is the claudin-1-KO mouse (Tokuda et al., 2014; Otani and Furuse, 2020), which dies in the perinatal period due to severe epidermal barrier defects and transdermal fluid loss (Furuse et al., 2002). An example of functional redundancy is provided by claudin-2 and claudin-15, both of which form intestinal cation and water channels. Mice lacking either claudin-2 or claudin-15 are viable. However, mice lacking both claudin-2 and claudin-15 die within a few weeks of birth, due to malabsorption (Wada et al., 2013). In Madin–Darby canine kidney (MDCK) II cells, claudin-1, -2, -3, -4 and -7 must all be knocked out in order to eliminate TJ strands and the paracellular barrier to ions; however, even then the barrier to much larger molecules is maintained unless JAM-A is also deleted (Otani and Furuse, 2020). These observations – as well as the phenotypes of JAM-A-KO mice, which display compromised intestinal and lung alveolar barrier function (Laukoetter et al., 2007; Mitchell et al., 2015; Hartmann et al., 2020) – show that JAM-A is a key component of the barrier to large molecules. The precise mechanisms by which this occurs are not yet fully defined but could include the recruitment of scaffolding proteins for other TJ membrane proteins and the regulation of signaling proteins by JAM-A. In contrast to KO of JAM-A, KO of either occludin or tricellulin does not compromise the macromolecular barrier in mice but does affect TJ permeability in cultured cell models. However, both tricellulin-KO mice and occludin-KO mice develop hearing loss, and occludin-KO mice show male sterility (Saitou et al., 2000; Nayak et al., 2013; Kitajiri et al., 2014; Kamitani et al., 2015), suggesting that occludin and tricellulin make significant contributions to cellular functions in specialized tissues, likely by enhancing TJ strand network complexity and numbers of branch points, and by connecting TJ strands to the central sealing element of tTJs.

## The role of TJs in epithelial polarity and intramembrane fence function

Epithelial apico-basal polarity is established through the concerted action of conserved apical polarity complexes (such as the Par complex and Crumbs complex) and lateral polarity complexes (such as the Scribble complex), as well as their downstream targets and effectors, which orchestrate cytoskeletal reorganization driven by Rho-family GTPases. Components of apical polarity complexes have been found to localize at the AJ, the TJ or the marginal zone, which is a Crumbs3 (CRB3)-containing region apical to the TJ and near to the free apical membrane (Martin et al., 2021). Moreover, several polarity complex proteins, including the Crumbs complex proteins PATJ and PALS1, and the Par complex protein Par3 (PARD3), have been shown to interact with TJ proteins, including ZO proteins and JAM-A. The Par complex is particularly important for the coordination of Rho GTPase signaling during the initial assembly of the AJC, and the PALS1–PATJ complex is important for tethering TJs to the marginal zone and expanding the TJ along the apical circumference (Martin et al., 2021; Groh et al., 2024).

The observation that TJs in mammalian cells reside at the border between the apical and basolateral membranes of epithelial cells has led to the concept that TJs serve as an intramembrane ‘fence’ to separate compositionally distinct apical and basolateral domains. However, MDCK II cells that lack all claudins, and hence do not have TJ strands, maintain normal apico-basal polarity of several apical and lateral protein markers. This demonstrates that TJ strands are dispensable for fence function. On the other hand, the complete loss of both ZO-1 and ZO-2 results in the disorganization, but not complete loss, of epithelial polarity, as indicated by altered distribution of the same marker proteins. This suggests that, although ZO proteins participate in the maintenance of apico-basal polarity, this is independent of their claudin-scaffolding functions, and that additional junctional complexes are implicated in the fence function of the AJC (Otani and Furuse, 2020). Thus, the precise molecular mechanisms and the identities of the protein and lipid components responsible for the fence function of the AJC remain to be determined.

## Cytoplasmic scaffolding and adaptor proteins

Beneath the TJ lies a cytoplasmic plaque of scaffolding and adaptor proteins that connects TJ transmembrane proteins to the actomyosin and microtubule cytoskeletons (Rouaud et al., 2020). This cytoplasmic domain is critical for TJ structure, function and regulation; a lack of scaffolding proteins results in loss of the TJ strands and permeability barrier. Most TJ scaffolding proteins [ZO-1, ZO-2, ZO-3, MUPP1 (MPDZ), MAGI-1 and a few others] contain PDZ domains that mediate their binding to the intracellular C-terminal cytoplasmic regions of transmembrane TJ proteins such as claudins and JAM-A. ZO-1 and ZO-2 are essential to redundantly promote claudin clustering and polymerization into TJ strands (Umeda et al., 2006). Among the adaptor and scaffolding proteins that do not contain PDZ domains are cingulin and paracingulin (cingulin-like-1, CGNL1; also known as JACOP, junction-associated coiled-coil protein), which bind to the C terminus of ZO-1 and contain a coiled-coil rod region that interacts with the coiled coils of specific isoforms of non-muscle myosin-2 (Vasileva et al., 2022; Rouaud et al., 2023). Functional redundancy among scaffolding and adaptor proteins, as well as the specific experimental model, cellular context and/or developmental stage, determine the relevance of scaffolding proteins for barrier function. For example, although the complete KO of ZO-1 in mice leads to an early embryonic-lethal phenotype (Katsuno et al., 2008), conditional KO of ZO-1 in the mouse intestine does not result in altered TJ structure and barrier function (Kuo et al., 2021).

Finally, biophysical processes involving scaffolding and adaptor TJ proteins also control TJ formation. For example, ZO-1 undergoes liquid–liquid phase separation (LLPS) during TJ formation in cultured cells and developing embryos, and is regulated by multimerization, multivalent interactions, mechanical force and dephosphorylation of ZO-1 (Beutel et al., 2019; Schwayer et al., 2019; Citi, 2020; Sun et al., 2022). Several additional TJ scaffolding and adaptor proteins are predicted to undergo LLPS based on the presence of intrinsically disordered domains (Rouaud et al., 2020). However, how the phase separation of different TJ molecular components is hierarchically coordinated in space and time is not clear.

## TJs as signaling hubs

Several TJ proteins, including JAM-A, ZO proteins, CGN and CGNL1, can recruit various signaling proteins to TJs. These include GEFs and GAPs for the Rho, Rab and Rap families of GTPases, as well as kinases, phosphatases and transcription factors (Citi et al., 2014; Zihni et al., 2016; Hartmann et al., 2020). Such signaling proteins have been implicated in the regulation of TJ assembly, disassembly and remodeling; barrier function; apico-basal morphogenesis; and cell proliferation, differentiation and migration in different experimental contexts (Higashi et al., 2016). For example, *in vitro* and *in vivo* experiments show that localized activation and inactivation of Rho and Rac GTPases regulates actin cytoskeleton remodeling during junction assembly and disassembly (Varadarajan et al., 2019). The recruitment of specific GEFs and GAPs by TJ proteins directs spatial remodeling of the actin cytoskeleton both at the AJC and elsewhere in the cell. Some signaling proteins are inactivated by sequestration at TJs. These include the transcription factors DbpA (also known as YBX3 or ZO-1-associated nucleic acid-binding protein, ZONAB), which redundantly binds to ZO-1 and ZO-2, and yes-associated protein 1 (YAP1) and TEA-domain family member 1 (TEAD1), which bind to ZO-2, as well as the RhoA activator GEF-H1 (also known as ARHGEF2), which binds to CGN (Aijaz et al., 2005; Citi et al., 2014; Spadaro et al., 2014; Gallego-Gutierrez et al., 2021; Balda and Matter, 2023). In this case, the release of these signaling proteins upon epithelial injury and/or junction disassembly provides a mechanism to initiate rapid transcriptional and cytoskeletal cellular responses.

## Association of TJs with the actomyosin cytoskeleton

**Fig. 3.**
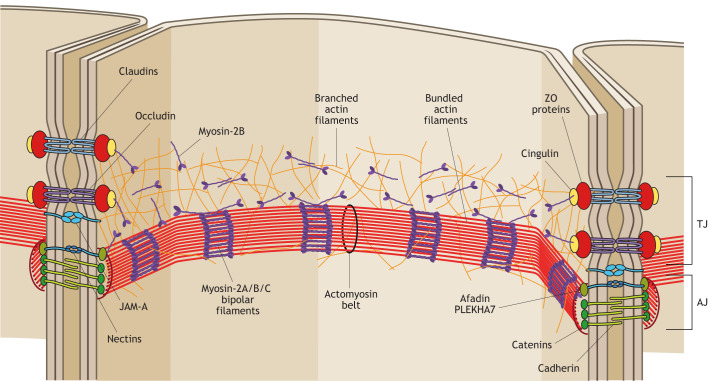
**A model of the architecture of the AJC.** Scheme showing a simplified model of the architectural organization of the AJC (bicellular junctions) and the associated actomyosin cytoskeleton. For simplicity, only a few major transmembrane proteins (occludin, claudins, JAM-A and cadherin), cytoplasmic scaffolds (ZO proteins), adaptors (cingulin) and cytoskeletal proteins (actin and myosin only, not microtubules) are shown. Occludin is representative of TAMPs, which include tricellulin at tTJs. JAM-A is representative of Ig-like CAMs, which include angulins at tTJs. Branched actin filaments associated with monomers or oligomers of myosin-2B are believed to tether the actomyosin belt to TJs, either by direct binding of actin filaments to ZO-1- and JAM-A-based complexes, and/or by cingulin-dependent tethering of myosin-2B to ZO-1. Proteins and protein complexes are not drawn to scale, and protein complexes are only shown at the section of membranes, whereas *in vivo* they are distributed continuously along the circumference of the AJC. This scheme was inspired by a similar scheme in Cartagena-Rivera et al. (2017), which is published under the terms of a CC-BY 4.0 license.

The actomyosin cytoskeleton, which forms a continuous circumferential belt at the AJC, plays a fundamental role in the architecture, assembly, remodeling and regulation of TJs. Initially observed by TEM as a region of dense cytoplasmic material adjacent to the TJ (Fig. 1A,B), the actomyosin cytoskeleton has subsequently been shown to be important for TJ function, as drugs that depolymerize actin filaments also disrupt TJ fibril structure and barrier function (Arnold et al., 2017). The perijunctional AJ-associated actomyosin belt contains parallel bundles of actin filaments associated with regularly stacked non-muscle myosin-2 bipolar filaments in a mini-sarcomere arrangement (Ebrahim et al., 2013) and is associated with E-cadherin (CDH1) at the AJ through catenin-containing (β-catenin and α-catenin) complexes (Fig. 3). Among the non-muscle myosin-2 isoforms expressed in TJ-containing cells (myosin-2A, myosin-2B and myosin-2C, which have heavy chains encoded by *MYH9*, *MYH10* and *MYH14*, respectively), myosin-2A has so far emerged as the most relevant isoform to provide the mechanical forces to regulate TJ assembly and function *in vitro* (Lechuga et al., 2023), together with myosin regulatory light chain (MLC) phosphorylation (Turner et al., 1997). Furthermore, mice with conditional KO of myosin-2A in the intestinal epithelium have a leaky epithelial barrier under homeostatic conditions and show exaggerated epithelial damage during mucosal inflammation, highlighting the concept that myosin-2A is a critical architectural component of the AJC (Naydenov et al., 2016). However, filaments containing myosin-2B and myosin-2C have also been found to localize at the circumferential actomyosin belt of the AJC, with some tissue-specific differences. Importantly, the connection of the actomyosin belt to the AJC occurs through a network of branched actin filaments associated with myosin-2B, which acts as a linker and force transducer to ZO-1 through cingulin (Fig. 3) (Heuze et al., 2019; Rouaud et al., 2023). The apical region of the ZA is characterized by the presence of nectin adhesion molecules and a complex comprising afadin and PLEKHA7 as cytoplasmic scaffold and adaptor proteins, whose localization is intermediate between ZO-1 and E-cadherin (Takai et al., 2008; Meng and Takeichi, 2009; Pulimeno et al., 2010; Shah et al., 2016; Rouaud et al., 2020; Mangeol et al., 2024) (Fig. 3).

Modulation of AJC-associated actomyosin belt contraction occurs through the phosphorylation of MLC, which tunes actin filament contraction to control barrier function in development, homeostasis and disease states such as inflammation. MLC can be directly phosphorylated by myosin light chain kinase (MLCK; encoded by *MYLK*) or by Rho kinases (ROCKs). In addition, ROCKs can increase MLC phosphorylation by acting on and inhibiting MLC phosphatase. Numerous *in vitro* studies have shown that activation of ROCKs can lead to TJ disruption and epithelial barrier loss (Quiros and Nusrat, 2014). Moreover, there is evidence that activation of RhoA by GEF-H1 is an important mechanism in enhancing barrier dysfunction caused by mechanical, chemical or pathological stimulation in endothelial cells (Balda and Matter, 2023). Finally, both *in vivo Xenopus* studies and *in vitro* analyses of mammalian epithelia show that activities of Rho GTPases and ROCKs are critical for the repair of junctional damage and for TJ remodeling to maintain barrier function (Varadarajan et al., 2019). Studies in *Xenopus* also show a role for mechanosensitive Ca^2+^ flashes and the RhoA GEF p115RhoGEF (also known as Arhgef1) in promoting RhoA activation to repair damaged TJs and thus maintaining barrier function (Varadarajan et al., 2019; Bement et al., 2024).

*In vitro* and *in vivo* studies show that MLCK-mediated MLC phosphorylation is the principal driver of TJ permeability triggered by physiological Na^+^–nutrient cotransport and by pathological exposure to cytokines such as tumor necrosis factor (TNF) (Berglund et al., 2001; Clayburgh et al., 2005; Yu et al., 2010). *In vivo*, either chemical inhibition or KO of the long MLCK splice variant, MLCK1, expressed in intestinal epithelium prevents pathogenic MLC phosphorylation, occludin endocytosis and barrier loss following immune activation. More recently, this has been shown to depend on recruitment of MLCK1 to the perijunctional actomyosin belt (He et al., 2020), which can be inhibited chemically. This is a remarkably effective therapy in a mouse model of experimental inflammatory bowel disease (Graham et al., 2019; Horowitz et al., 2023; Zuo et al., 2023).

Specific cytoplasmic scaffolding and adaptor proteins of TJs crosstalk with the actomyosin cytoskeleton in different manners, for example by mediating the connection of actin filaments, non-muscle myosin-2 and associated proteins to the TJ membrane. The C-terminal half of ZO-1 comprises an actin-binding region that interacts with low affinity with actin filaments, and this weak link is required for dynamic remodeling of TJs and barrier function *in vitro* (Yu et al., 2010; Belardi et al., 2020). ZO-1 also contains a C-terminal ZU5 domain, which interacts with the N termini of CGN and CGNL1, which in turn contribute to tethering myosin-2B to the AJC. This connection regulates TJ membrane tortuosity, apical membrane stiffness and AJ organization without, however, affecting the epithelial TJ barrier *in vitro* (Vasileva et al., 2022; Rouaud et al., 2023). In addition, ZO-1 is a mechanosensing protein, and the connection of ZO-1 to myosin-2B through CGN promotes the stretched conformation of ZO-1, which in turn is required *in vitro* for ZO-1 binding to ligands such as occludin and DbpA, and for ZO-1 LLPS and accumulation at TJs (Spadaro et al., 2017; Beutel et al., 2019; Vasileva et al., 2022). Simultaneous deletion of claudins, JAM-A and CAR affects the nanometer-scale organization of ZO-1, and leads to disruption of the AJC and the underlying actin filaments upon spontaneous cell stretching (Nguyen et al., 2024). These results suggest that the TJ membrane proteins play a crucial role in the integrity of the AJC by regulating the nanoscale organization of ZO-1 near the TJ membrane. Recently, it has been shown that ZO-1 interacts with COBL, which is an actin- and microtubule-interacting protein that is capable of inducing LLPS and acts as a nucleator for actin polymerization, promoting the formation of actin filament bundles at TJs and AJs (Tsukita et al., 2023).

## Association of TJs with the microtubule cytoskeleton

Microtubules are required for the trafficking of TJ transmembrane proteins, and their polymerization is particularly important for endothelial TJ barrier function, due to the ability of polymerized microtubules to sequester regulators of Rho-family GTPases, such as GEF-H1 (Karki and Birukova, 2021). Several TJ proteins organize microtubules at TJs. For example, CGN interacts directly with microtubules, CGNL1 tethers microtubule minus ends to TJs by recruiting the microtubule minus-end-binding protein calmodulin-regulated spectrin-associated protein 3 (CAMSAP3) to TJs, and both CGN and CGNL1 organize the planar apical network of microtubules in cultured Eph4 cells (Yano et al., 2013; Flinois et al., 2024). Depletion of ZO-2 also alters the cytoarchitecture and organization of microtubules in MDCK II cells (Raya-Sandino et al., 2017). *In vivo*, KO of CGNL1 in mice results in disorganized apico-basal positioning of nuclei and disorganized microtubule architecture in the intestinal epithelium. Moreover, leucine zipper protein 1 (LUZP1) binds to both microtubules and ZO-1 and promotes apical constriction of epithelial cells by inhibiting MLC phosphatase (Yano et al., 2021). The interaction of TJ proteins with microtubules or microtubule-binding proteins regulates the distribution and apical organization of microtubules and can affect epithelial polarized architecture, but its role in regulation of TJ barrier function is unclear.

## TJs in disease and as drug targets

TJs are implicated in a variety of diseases and can be manipulated to perturb barrier function and aid drug delivery. Studies of mouse models have shown that claudins are involved in several pathological processes, including inflammation, cancer, neurological diseases, metabolic disorders and infections (Mitic et al., 2000; Furuse, 2009; Tsukita et al., 2019; Piontek et al., 2020). For example, mice in which the expression of claudin-1 is knocked down, or in which claudin-2, -7 or -18.2 are knocked out, show inflammatory phenotypes in the skin (atopic dermatitis), small intestine, large intestine and stomach, respectively. KO studies further indicate that claudin-18.1 and claudin-18.2 play a role in tumorigenesis in the lung and stomach, respectively (Hagen et al., 2018), and claudin-18.1 is also implicated in infection-related processes. Intestinal claudin-15 KO leads to malabsorption, and simultaneous KO of claudin-2 and claudin-15 results in severe malnutrition and death. The onset of gallstones is promoted by KO of claudin-2 and claudin-3. Furthermore, many metabolic disorder pathologies in the kidneys are identified following KO of claudin-2, claudin-8, claudin-10a and claudin-16 (Meoli and Günzel, 2023).

Altered expression of specific TJ proteins has been detected in many cancer types, and TJ proteins have been identified as potential mediators of resistance to apoptosis and anoikis, cancer stemness-like phenotype acquisition, and migration and plasticity of cancer cells (Gonzalez-Mariscal et al., 2016; Kuo et al., 2019, 2021; Nehme et al., 2023). Moreover, mutations in the genes encoding claudins have been identified in several hereditary diseases. For example, the hereditary absence of claudin-1 in humans triggers neonatal ichthyosis and sclerosing cholangitis (NISCH) syndrome. Mutations in claudin-5, the endothelium-specific claudin isoform, lead to impaired neuronal development and function, altered blood–brain barrier function, seizures, microcephaly and brain calcifications (Deshwar et al., 2023). Mutations in additional TJ proteins, including tricellulin, claudin-14 and cingulin, as well as genomic duplication of ZO-2, are also associated with hereditary forms of deafness (Krug et al., 2014; Zhu et al., 2023).

As mentioned above, TJ barrier function is perturbed in inflammatory bowel diseases, thereby contributing to disease pathogenesis, through multiple actomyosin-dependent mechanisms (France and Turner, 2017; Buckley and Turner, 2018). In addition, there are cytoskeleton-independent mechanisms through which TJ barrier function is perturbed in inflammatory bowel diseases. These include downregulation of barrier-forming claudins, tricellulin and JAM-A, and upregulation of channel-forming claudins such as claudin-2 by proinflammatory cytokines (Luissint et al., 2016; Krug et al., 2018). An active area of research addresses how to repair TJ barrier function in inflammatory bowel disease through reduction of the cytokine-induced contractility of the actomyosin cytoskeleton (Horowitz et al., 2023). Conversely, temporary loosening of the TJ barrier would be very useful to allow the delivery of drugs across tissue barriers, such as the blood–brain barrier, and to enhance fluid efflux, which promotes intestinal pathogen clearance. Several small molecules, peptides, antibodies and other reagents have been developed that directly or indirectly target TJ components, mostly transmembrane proteins, to modulate TJ organization, barrier function, and TJ-related cytoskeletal remodeling and signaling (Ramirez-Velez and Belardi, 2023). Finally, it must be noted that some transmembrane TJ proteins act as cell-entry factors for viruses, including hepatitis C virus (HCV), reovirus, coxsackievirus and adenovirus, West Nile virus, and rotavirus. HCV is a major cause of liver cancer, and treatment of patient-derived cancer cells with monoclonal antibodies against claudin-1, the receptor for HCV, suppresses liver cancer growth in *ex vivo* models (Nehme et al., 2023). The functional analysis of these molecules will potentially unveil new aspects of claudin functions.

In summary, TJ involvement in a broad range of diseases provides hope that synergy between recent basic and translational advances will lead to the development of new approaches to treat disease.

## Conclusions and challenges for future studies

TJs that form impermeable or selectively permeable paracellular barriers are critical to separate tissue and body compartments within multicellular organisms. In addition to providing barrier function, TJs participate in signaling to regulate epithelial cell morphogenesis and behavior. TJs are the only definitive structural entities that form epithelial paracellular barriers, and we believe that the term TJ should ideally be restricted to junctions that fulfil the specific ultrastructural, functional, architectural and molecular criteria listed in Box 1. Other terms, such as septate junctions and TJ-like junctions, should be used to define junctions that are found in invertebrate organisms and in other specialized vertebrate contexts but that do not share the key properties of ‘canonical’ TJs.

Our understanding of TJs at the molecular level has made huge strides since the first identification of zonulae occludentes by Farquhar and Palade over 60 years ago. Within 25 years (1986–2011), most protein components of TJs were discovered, initially through the generation of specific antibodies, and subsequently through the use of biochemical, proteomic and genomic tools. More recently, improved imaging, structural, molecular and biophysical approaches have provided insights that were unimaginable even only a decade ago. However, many important questions remain open, and essential molecular details are missing. The three-dimensional structures of most TJ proteins, either alone or in complexes, remain to be determined. More importantly, the structure of these proteins within cells *in situ*, and how interactions with other proteins, architectural constraints, LLPS and mechanical forces affect their organization and conformation is not known. The biophysical and biochemical principles guiding the step-by-step development of the architectural organization of the AJC are also not well understood. Although the functions of many TJ proteins are beginning to be clarified through the use of KO and knock-down models, there are still large gaps in our understanding of the diverse roles of these proteins in specific cell, tissue and developmental contexts. Much of our current understanding of TJ protein function and regulation is based on *in vitro* studies employing a few cellular model systems. Validation of these functions will require extensive work *in vivo* in different animal models, tissues and cell types, and under physiological conditions and pathological stress. Progress in these areas, and the identification of novel hereditary diseases resulting from mutations in genes encoding TJ proteins, will be essential to expand the possibility of exploiting TJs and their proteins as drug targets and diagnostic tools. Finally, further investigations into the biochemical composition and functional organization of TJ-like junctions in invertebrate phyla will help us to develop a deeper understanding of the evolution of barrier-forming junctions, from sea sponges to mice and humans.
